# Physico-Mechanical Characteristics of Gypsum–Fiber Boards Manufactured with Hydrophobically Impregnated Fibers

**DOI:** 10.3390/ma17184555

**Published:** 2024-09-17

**Authors:** Adrian Trociński, Dorota Dziurka, Marta Thomas, Radosław Mirski

**Affiliations:** 1 Department of Mechanical Wood Technology, Poznań University of Life Sciences, ul. Wojska Polskiego 28, 60-627 Poznań, Poland; adrian.trocinski@up.poznan.pl (A.T.); dorota.dziurka@up.poznan.pl (D.D.); 2 Faculty of Civil and Transport Engineering, Poznan University of Technology, Piotrowo 5, 60-965 Poznań, Poland; marta.thomas@put.poznan.pl

**Keywords:** gypsum boards, hemp fibers, impregnation

## Abstract

Although gypsum-based building materials exhibit many positive characteristics, solutions are still being searched for to reduce the use of gypsum or improve the physico-mechanical properties of board materials. In this study, an attempt was made to produce gypsum boards with hemp fibers. Although hemp fibers can be a specific reinforcement for gypsum-based board materials, they negatively affect the gypsum setting process due to their hygroscopic characteristics. Fibers impregnated with derivatives based on polyvinyl acetate, styrene–acrylic copolymer and pMDI (polymeric diphenylmethane diisocyanate) were used in this study. Gypsum–fiber boards produced with impregnated fibers showed approximately 30% higher mechanical properties as determined by the 3-point bending test. The positive effect of the impregnates on the hemp fibers was confirmed by FTIR (Fourier-transform infrared spectroscopy) and TG/DTA (thermogravimetric analysis/thermal gravimetric analysis) analysis.

## 1. Introduction

Gypsum is a naturally occurring mineral from the sulfate group. Around 70 different forms of gypsum are known. Its crystals often fuse to take various forms and are known by other names. It is widely extracted in Canada, Chile, the United States, Russia, Italy, Germany and Poland. Gypsum is a karstic rock in which caves are formed. The most exciting caves of this type are found in Podolia. Man has used it for thousands of years for its ease of obtaining and processing. It is mainly used in art and sculpture [1,2,3,4,5] and in the construction industry in the broadest sense of the term, which includes both road building [6,7,8,9,10] and admixtures for cement or other building materials [11,12,13]. However, the gypsum board is one of the most recognizable gypsum products in the construction industry [14,15,16]. There are 160 gypsum and plasterboard factories in Europe, employing more than 28,000 people. The turnover exceeds EUR 7.7 billion [17]. Plasterboard is primarily used as wall cladding in traditional construction and wherever fire resistance requirements are increased. Undoubtedly, the high fire resistance of gypsum board coatings is due to the gypsum itself and the board structure limiting the movement of fire or heat to deeper layers. Structures covered with gypsum boards fail because of deformations and deformations of the load-bearing part, not the sheathing. For this reason, numerous studies have been conducted to assess phenomena, carried out empirically and numerically [18,19,20,21]. It is estimated that, due to the endothermic dehydration process of gypsum occurring at high temperatures, these panels can maintain sufficiently high fire resistance ratings. By introducing lignocellulosic material into the structure of gypsum boards, the fire resistance of the boards can be compromised to some extent. Still, at the same time, thermal insulation and mechanical strength can be improved. This reasoning may be consistent with the findings of Wang et al. [22], who improved the fire resistance of reduced-density gypsum boards by using palygorskite and cost-effective glass fiber.

Gypsum-based boards can also contain organic particles that fulfill different roles. Deng and Furuno [23] proposed the introduction of polypropylene fibers into the gypsum paste during panel manufacture. The length and amount of fibers added significantly affected the boards’ tensile strength, as well as the modulus of elasticity at fracture. They further showed that fibers of 9–12 mm in length, introduced at 9–12%, had a beneficial effect on mechanical properties. On the other hand, Yalcin and Kaya [24] showed that it is possible to produce plasterboards containing wood chips (*Pinus brutia*), with characteristics that allow them to be used in less demanding areas. The thermal properties of the manufactured panels were improved, while the mechanical properties were weakened. The authors suggest that less than 20–30% of wood particles by weight should be introduced to achieve acceptable board quality. Other studies suggest that the poorer quality of these boards is due to the poor bonding of the gypsum to the wood, which can be improved by adding cement to the system [23,25,26]. It is easier to introduce plant material into systems containing cement, such as sawdust, pomace, hulls, or even paper waste [27,28,29]. However, combining gypsum with cement is less advisable in the case of plasterboard. Similar problems, but this time with fibers bonding to gypsum, were demonstrated in their work by Abir and his team [30]. They produced gypsum boards with jute fibers. Most of the mechanical properties of the boards produced deteriorated. However, an improvement in their thermal properties was observed. FTIR analysis demonstrated the absence of chemical changes in the gypsum–fiber system, confirming that gypsum has no chemical affinity with lignocellulosic materials. They point to a purely mechanical bonding effect. The problem of poor adhesion to lignocellulosic materials can be minimized by impregnating them with adhesion-enhancing agents. For example, Deng and his team [31] used citric acid for this purpose. They found that wood particles impregnated in this way, combined with gypsum, made producing boards with satisfactory properties possible. Admittedly, the structure of wood chips differs from that of fibers, but this concept seems valid. 

Therefore, this study aimed to evaluate the possibility of impregnating hemp fibers with adhesion promoters to improve the adhesion of gypsum to fibers.

## 2. Materials and Methods

Building gypsum obtained from Dolina Nidy (Leszcze, Poland) was used in the study. It is a gypsum of natural origin obtained from open-pit mines in southern Poland. The feature that distinguishes building gypsum from other gypsum masses is its relatively fast setting time. In general, building gypsum is the starting material for producing gypsum putties, adhesives and plaster joints, which are made by introducing specialized mineral fillers and modifying agents into the basic form. Table 1 gives the essential characteristics of the gypsum used in the study, as specified by the manufacturer.

**Figure 1:**
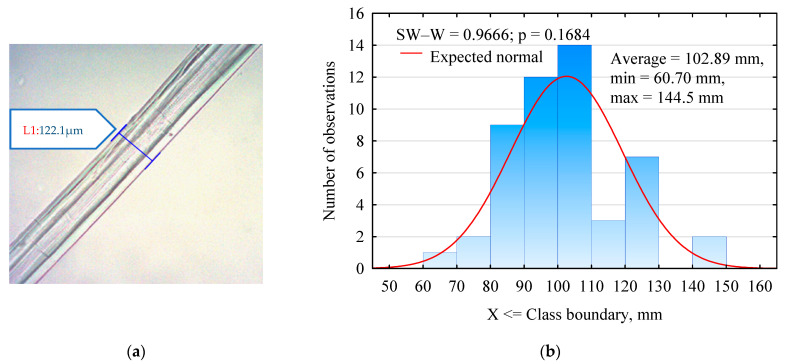
(**a**) Photo of a hemp fiber taken with a microscope camera; (**b**) histogram of the thickness of the hemp fibers used in the study.

It was decided to reinforce the gypsum matrix with fibers from hemp seed (*Cannabis sativa* L.) of the Białobrzeska variety, grown in the Wielkopolska region. The fibers were obtained as pure fibers, hydraulically cut by the manufacturer to a specific length, i.e., 5 mm, 20 mm, 50 mm and 100 mm. The fibers were supplied by the LenKon company (Stęszew, Poland), which is also the experimental facility of the Institute of Natural Fibers and Herbaceous Plants in Poznań (Poland). The fibers used in the study were subjected to thickness assessment by measuring the diameter of the fiber at mid-length. Measurements were made using a Moticam 10+ microscope camera with dedicated Images Plus 3.0 PL software (Figure 1). The average fiber thickness was 117 μm, with a standard deviation of 7.12 μm. The thickness range, however, was from 102 μm to 128 μm (min. and max. values). Despite the different measurement methods, growth location and subspecies, the fiber thickness measurements obtained are within the range of 109–134 μm [32].

Since hemp fibers, like most lignocellulosic materials, are hygroscopic and swell enormously, absorbing water to the point of fiber saturation, it was decided to reduce their natural susceptibility to water absorption. Hemp fibers have no chemical affinity with gypsum, so their anchoring process relies mainly on mechanical adhesion or the ability to resist the mutual displacement of the fibers and the surrounding gypsum. This process can occur when the shrinking plaster ‘tightens’ on the fibers due to binding.

Unfortunately, the fibers that donate water to the gypsum hydration process will lose their linear dimensions, thus limiting the ‘strength’ of the process. For this reason, it was decided to modify the surface of the hemp fibers with the following products used in the construction or wood industry:

- Deeply penetrating primer (Soudaprim NF) manufactured by Soudal. The manufacturer indicates that it is a preparation used to strengthen the surface of partitions before applying other layers and that it reduces the absorption of both mineral and organic materials. The main component of this preparation is an aqueous solution of polyacetylvinyl. It also contains various plasticizers and liquefying and preservative additives. The primer is designated S (Soudal).
- A universal priming emulsion produced by Unicell for the Castorama chain of companies. The product is designed to protect interior surfaces. The manufacturer indicates that the layer created on the surface is resistant to water and alkalis, increasing the adhesion of various types of gypsum to the covered surface and strongly reducing the substrate’s absorption. The emulsion is an aqueous solution of a styrene–acrylic copolymer with a pH of 7.0–9.0 and high fire resistance. The emulsion is designated U (Unicell).
- pMDI (methylenediphenyl diisocyanate MDI), which was chosen for the study because of its ease of bonding to a variety of materials containing active hydrogen atoms, and it has high water and temperature resistance. The adhesive was purchased from Bayer AG, Berlin, Germany. The adhesive is a mixture of three isomers with a density of approximately 1.12 g/cm^3^, a viscosity of 215 MPa-s at 25 °C and a pH of 6.5. According to the manufacturer, the adhesive contained 96 mg of hydrolytic chlorine and 30.9% active NCO groups.

The modification process of the fibers consisted of their free saturation in soil solutions. The process was carried out at room temperature for 12 h. The solution containing the fibers was then filtered through a Schott filter, and the fibers were dried at 102 °C for 24 h. 

The pMDI glue was applied to the hemp fibers by air spray using a spray gun with a nozzle diameter of 1.3 mm. The amount of glue was 10% relative to the dry weight of the fibers. The fibers, together with the applied adhesive, were then subjected to drying at 102 °C for 24 h, taking care to ensure that they did not stick together. Regardless of the application method, the dried fibers were protected from moist air until they were used. 

The fibers were subjected to an assessment of moisture shrinkage in length and wettability to check the effectiveness of the impregnates used. 

The moisture shrinkage of hemp fibers was determined after a 24 h fibers bath in water at room temperature. Twenty-five fibers, randomly selected from the delivered batch, were tested. The hemp fibers at its top were fixed to the base with an entomological pin (Ø 0.27 mm), then stretched and pressed against the base glass. The first measurement was taken immediately after the above steps, considering it to be the length of the fibers in their thoroughly wet state. The next stage of the test was to direct a heat source (25 V incandescent lamp) to the measurement site to increase the fibers’ drying intensity. The second measurement was carried out one hour after the start, and subsequent measurements were taken every 10 min until three equal measurements were obtained, which indicated that the fibers had been brought to a dehydrated state. A Sylvas2tic electronic caliper (Sylvac S.A, Valbirse, Switzerland) was used for the measurement.

The test to determine the absorbency of the hemp fibers began by drying each variant at 102 ± 2 °C. The dried fibers were divided into 5 ± 0.5 g batches and placed in separate laboratory beakers. The start of the measurement time was considered to be when water was introduced into the beakers with the fibers. Fiber saturation was measured after the following periods, from the start of the process: 15 min, 30 min, 60 min, 120 min and 300 min. After a given time, the fiber mixture was poured into a Schott crucible, and the water was pressure-drained. The mass obtained on the sieve was drying at 102 °C for 24 h. The difference in masses before and after drying was used to determine the amount of water absorbed by the fibers.

**Figure 2:**
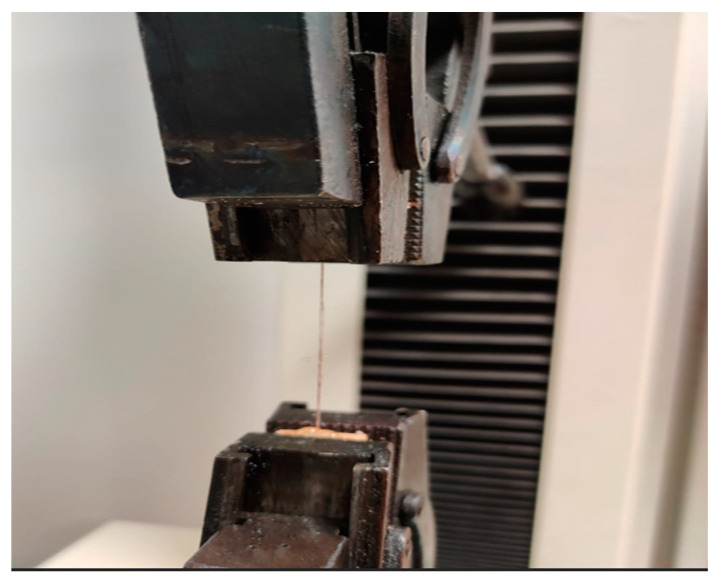
Fiber in the handles of the testing machine.

They were subjected to a tensile strength assessment to assess the mechanical properties of the fibers before and after impregnation. Individual fibers were sandwiched between fiberboard facings to fix the fibers in the grips of the tensile testing machine. The test stand is shown in Figure 2.

**Figure 3:**
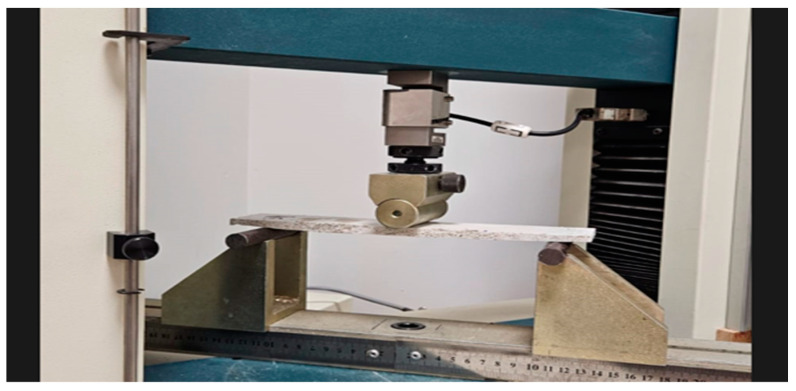
Measurement of mechanical characteristics in the 3-point bending test.

Plasterboard formats were produced using impregnated fibers. Two board variants were prepared. The primers were mixed with building gypsum in the first, according to a W-G ratio of 0.6l–1 kg. In the second, however, the fibers were impregnated in each of the tested primers for 12 h, after which they were dried to a moisture content of approximately 4%. The content of hemp fibers introduced in each case was 2%. The first variant was marked as A, and the second was B. Samples for static bending strength and modulus of elasticity tests were obtained from the panels and thus produced. The tests were carried out in a 3-point bending scheme on specimens 250 mm long, 50 mm wide and 10 mm thick. A photo of the mechanical characteristics test is shown in Figure 3. The produced panels were also subjected to FTIR and TG evaluation.

FTIR spectra of the analyzed variants were recorded using a Nicolet iS5 Fourier transform spectrophotometer(LPP Equipment AG, Uster, Switzerland) in the 4000–400 cm^−1^ range with a resolution of 4 cm^−1^. The thermogravimetric analysis (TGA) was carried out using a Netzsch STA 449 F5 Jupiter (Netzsch, Selb, Germany). The analysis was carried out with the following parameters: sample mass 20 ± 1 mg, atmosphere He, flow rate 15 mL/min, heating rate 5 °C/min, fixed final temperature 600 °C.

The study results were subjected to statistical analysis using Statistica 13.5 software. The results were subjected to ANOVA analysis of variance and post hoc tests (search for homogeneous groups with NIR test). The mean and standard deviation (SD) were used to analyze the results.

## 3. Results

**Figure 4:**
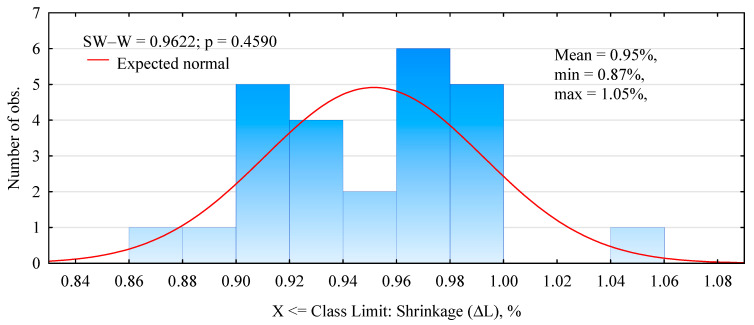
Histogram of moisture shrinkage along the length of hemp fibers.

The test results for changes in length after drying are shown in Figure 4. (The initial moisture content of the fibers was about 168%, after drying, about 1%). The shrinkage value tested ranges from 0.87% to 1.05%, with an average value of 0.94%. Knowing the characteristics presented allows the length differences within the composite to be determined. For the longest fibers used in the mechanical characterization tests (approx. 100 mm), the length difference due to moisture changes is approximately 1 mm (1%). The shorter the fibers, the smaller this difference is even more. Because the length shrinkage of hemp fibers is so insignificant, with short fibers of about 5 mm, such shrinkage may not significantly affect the mechanical properties of the gypsum–fiber composites produced. With long fibers, however, such changes may already have a significant effect. This is because building gypsums are characterized by a lack of contraction [21], which, in the case of fiber shrinkage, can cause voids in the structure of the composite and thus weaken it in terms of mechanical properties.

**Figure 5:**
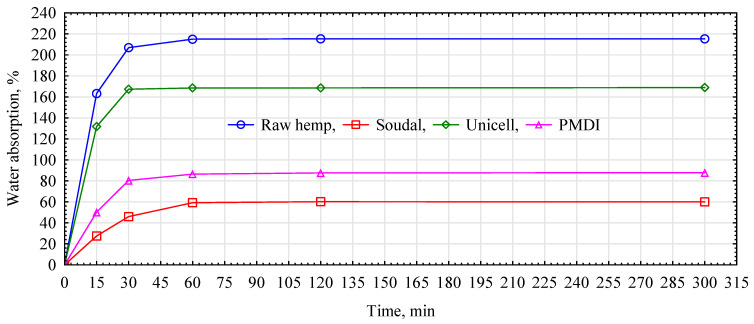
Absorbability of natural and modified hemp fibers.

Hemp fibers, like wood fibers, are hydrophilic materials. This phenomenon is attributed to the chemical structure of the material under study. The hydrophilicity of cellulose materials is due to the large number of -OH groups in its chains and the amorphous areas between the crystallites [33,34]. This phenomenon is important when hemp fibers are combined with gypsum binders, especially when the fibers are introduced into the slurry. The fibers can take away some of the binder water and thus affect the quality (strength) of the fiber–gypsum board produced, the ability to be easily formed in the matrix and the time at which the setting occurs. Methods, mainly chemical, affecting the hydrophilization of hemp fibers are indicated in the literature [35,36]. The choice of a fiber-modifying compound depends primarily on the matrix into which they are introduced. In the search for solutions that can influence even short-term reduction in fiber absorption while not degrading their surface and being neutral to the gypsum bond, the focus was on commonly available priming emulsions and pMDI adhesive. The results obtained from the test carried out in this regard are presented in Figure 5.

**Figure 6:**
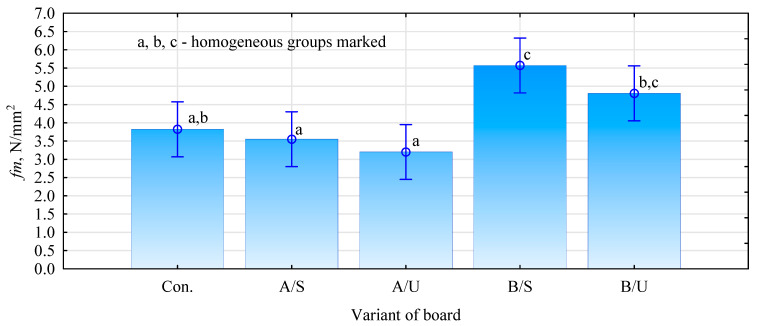
Flexural strength of fabricated composites.

Hemp fiber is characterized by high absorbability. The course of water absorption is very dynamic for the first 15 min, during which the moisture content of the fiber increases by more than 160%. After 30 min, the hemp fibers have more than doubled in weight compared to their dehydrated state. A further increase in absorbability, although already much smaller, is noticeable up to the first hour of the test, reaching a maximum value of 215%. For the next four hours, the fiber takes up no more water. The high and rapid absorption of hemp fibers, especially during the first stage of their contact with water, is a problematic factor, as this is when the gypsum bond formation and curing occurs. Regardless of the modifier used, hemp fibers only take up water during the first hour of the soak test. Fibers modified with Unicell primer increase in mass by 132% in the first 15 min. Thus, the agent reduced the absorbability of fibers modified with it, relative to raw fibers, by 31%. After 30 min, the Unicell-modified fibers had increased in mass by 167% relative to the dry fiber. Further measurements showed that the increase in absorbability of the hemp fibers reached 168%, after which it did not change significantly until the end of the test. The difference in absorbability between the maximum values was 46.5%. Hemp fibers treated with surface modification with pMDI adhesive and Soudal primer have significantly lower absorbability. When the fibers were impregnated with pMDI glue, the fiber mass increased by 50% for the first 15 min, after which the water uptake dynamics started to decrease. After 30 min, the fibers increased their mass by 80%. At further stages of the test, the fibers increased their mass to approximately 87% and, with slight variations (+0.2%), maintained this absorbability level until the test’s end. Fibers impregnated in pMDI are stiff and difficult to spread in the gypsum slurry after cross-linking the adhesive. Despite attempts, producing a composite with such impregnated fibers was impossible. Fibers impregnated with Soudal primer show the best quality characteristics. During the first fifteen minutes of soaking, the weight of the fibers increased from the dry state by only 27.5%. The difference in absorbability between the impregnated and raw fibers at this test stage (15 min) is almost 136%. After 30 min, the fibers had increased their mass by 46%, and after 60 min, they had reached their maximum absorbability of only 60%. The first 30 min of soaking is the most significant in the context of fiber–gypsum board manufacture, as during this time, most of the water evaporates from the slurry, and the fibers do not take up significant amounts of the make-up water, which can affect several properties of the final product. The saturated fiber during the board setting process can also affect the bonded gypsum matrix. The positive effect of the modification used was confirmed by tests to evaluate the mechanical properties (Figure 6). The panels produced by mixing the primers with the dry gypsum mass and introducing hemp fibers into the so-formed dense matrix, irrespective of the type of primer used, show a lower flexural strength (Figure 4). The boards manufactured with Soudal-impregnated fibers have a 9.8% lower strength than the reference variant. In contrast, the decrease is even more significant in the case of boards made with Unicell-impregnated fibers, for which the flexural strength decreases by almost 20%. Much better results were obtained by preparing the boards according to variant B. In this case, the boards produced with Soudal primer-impregnated fibers show an increase in strength of up to 40%, while those impregnated with Unicell show an increase of almost 21%. The differences between these variants are not statistically significant.

**Figure 7:**
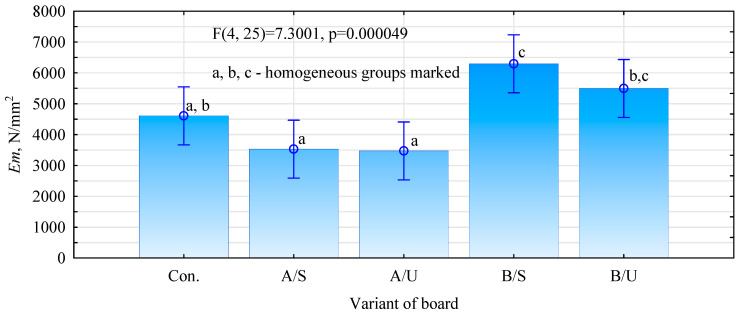
Modulus of elasticity of produced composites.

Two mechanisms may have contributed to this. The first relates to a reduction in absorbability of the fibers and, thus, a lower uptake of the batch water by the hemp fibers. The lower increase in static flexural strength of panels with Unicell-impregnated fibers may be related to the poorer absorbability of these fibers compared to Soudal-impregnated fibers. The reduction in absorbability of the fibers may limit the rapid absorption of water from gypsum slurry and reduce the amount of water vapor given off by the fiber in the already cured gypsum matrix. This also is confirmed by the Soudal-impregnated fiber board variants, which reduce the absorbability of the fibers to a greater extent relative to non-impregnated fibers. The second mechanism may relate to the mechanical anchoring of the gypsum slurry. This phenomenon involves the gypsum slurry flowing into irregularities (suitably rough surfaces), forming anchorages or hooks [37]. The moduli of elasticity of the produced panels are proportional to the static bending strength results (Figure 7). The variant with fibers impregnated with Soudal and then dried before incorporation into the matrix shows the highest increase in modulus of elasticity values. In this case, the difference from the reference variant was 36.6%. It should also be noted that this method of impregnating the fibers also increased the board’s stiffness in the case of modification with Unicell by 19.3% compared to the reference variant. The analyses, therefore, show that not only the change in absorbability of the fibers has a significant impact on the mechanical quality of the panels produced but also how the lignocellulosic filler is introduced. Significantly, the industrial gypsum boards (samples were tested without external paper) show a relatively low static bending strength of 2.43 N/mm^2^ (SD = 0.29 N/mm^2^), i.e., more than two times lower than the most favorable variants and a relatively low modulus of elasticity of 1100 N/mm^2^ (SD = 89 N/mm^2^). The laboratory boards, therefore, show a marked increase in the mechanical properties determined by the bending test.

**Figure 8:**
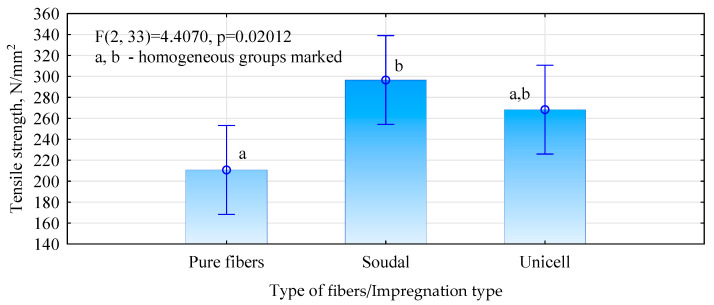
Tensile strength of hemp fibers modified and not modified with adhesion promoters.

To assess the influence of the impregnated fibers’ quality on the hemp-fiber boards’ strength, tests were carried out on the axial tensile strength of the fibers (Figure 8). The tests show that some increase in fiber strength is observed after impregnation. This increase is similar to the rise in the strength of the boards. However, the scatter in the results is very large. For this reason, there are no statistically significant differences between pure and Unicell-impregnated fibers and between Unicell-impregnated and Soudal-impregnated fibers. The increase in board quality can be attributed to an improvement in the load-bearing capacity of the fibers themselves. Still, we currently have too little information to assume that this is the only reason for the increase in the mechanical properties of the boards. However, our results for pure fibers are significantly lower than those reported in the literature [38]. 

**Figure 9:**
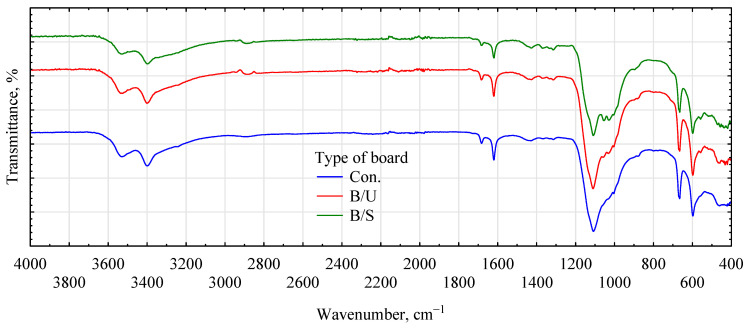
FTIR spectra of fiber–gypsum composites based on building gypsum.

FTIR spectra of samples of the respective gypsum–fiber boards were performed to confirm whether the selected primers actually fulfill the chemical or mechanical role attributed to them. The interpretation was based on the bands at 1000 and 1100/1150 cm^−1^, which are bands characteristic of gypsum (gypsum absorption band). In Figure 9, it is characterized by its high intensity and specific shape. In the case of the spectra of samples made of building gypsum and hemp fibers, two smaller bands emerge at the slope of this band, especially when Soudal priming emulsion is used. Peaks at 1300 cm^−1^ also appear weaker than expected but more pronounced than in the control board and in the Unicell-modified fiber boards at 2930 cm^−1^, associated with styrene–acrylic copolymer derivatives.

**Figure 10:**
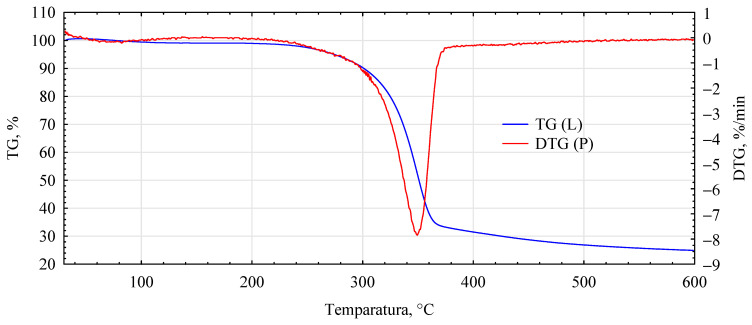
DTG analysis for unmodified hemp fibers.

**Figure 11:**
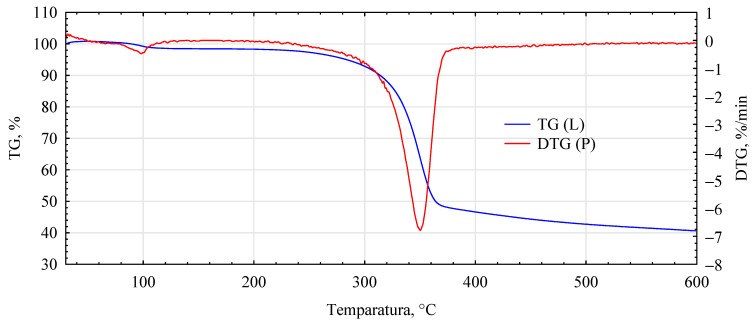
DTG analysis for fiber–gypsum composite.

**Figure 12:**
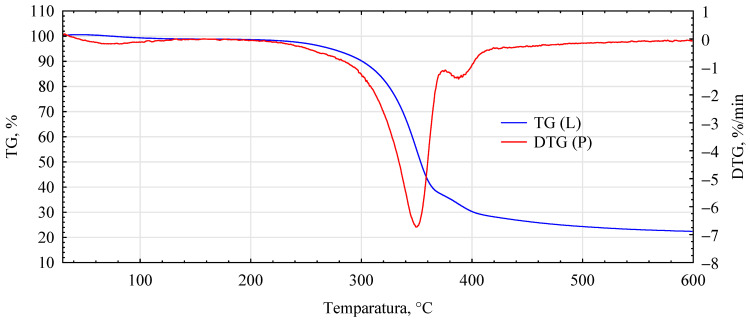
DTG analysis for hemp fibers modified with Unicell.

**Figure 13:**
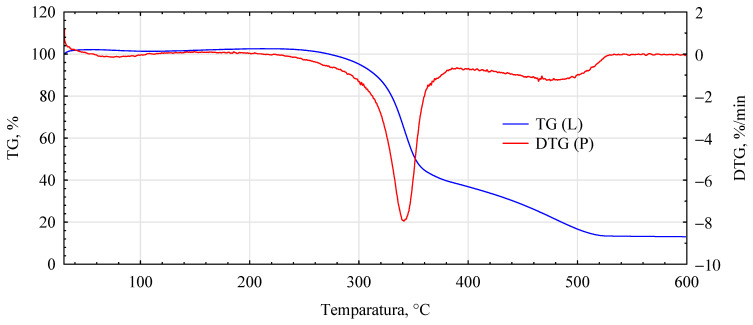
DTG analysis for hemp fibers modified with Soudal.

**Figure 14:**
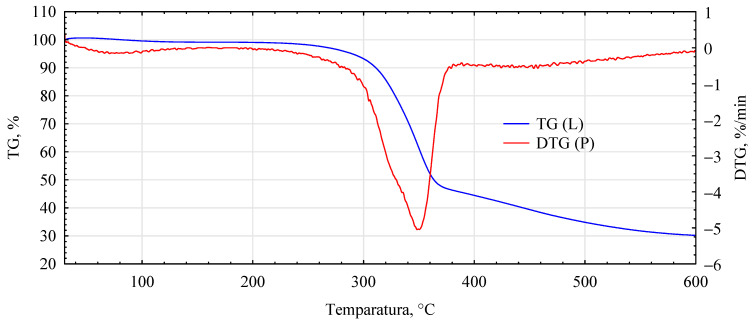
DTG analysis for hemp fibers modified with pMDI.

The influence of the introduced adhesion promoters is even more evident in the spectra of the thermal analyses. Similarities for the analyzed variants are only found in the initial heating phase, i.e., in the temperature range 40–150 °C (Figure 10, Figure 11, Figure 12, Figure 13 and Figure 14, Table 2). This area is generally considered to be the region where evaporation of water and low-molecular-weight volatile products takes place. Except for systems containing primers, only water evaporation is expected to have occurred in this area. Since systems containing primers show lower losses than pure fibers or fiber–gypsum board, evaporation of significant amounts of components other than water is not to be expected. Significant differences, therefore, mainly occur in the temperature range of 150–400 °C, especially 200–500 °C. The start temperatures of the transformation processes are relatively similar and start at 185 ± 1 °C for pure fiber and fiber impregnated with Soudal and gypsum–fiber board. Fibers impregnated with Unicell and pMDI show lower transition initiation temperatures. In this case, this temperature is about 10 °C lower at 175 ± 2 °C. It can, therefore, be assumed that in these two cases, decompositions other than essentially only hemp fibers may occur [39]. The length of the transformation region is similar for all systems. The maximum for this peak is at around 349 ± 1 °C and is not influenced by the process initiation temperature.

In contrast, the speed of decomposition varies. Thus, the fiber decomposes in this area at a 7.84%/min rate. This is slightly slower than the fibers impregnated with primer Soudal decomposition. In contrast, the fibers impregnated with pMDI decompose the slowest. This is reflected in the previously reported positive effect of pMDI on the characteristics of the fibers impregnated in this way in terms of absorbability.

Furthermore, the total weight loss for this area is only lower for the fiber placed in the gypsum shell. The priming impregnates used in the study have different effects on the fiber distribution in this temperature region. In the case of primer one, both the onset of transformation temperature and the transformation rate are relatively low, lower than that recorded for pure fiber. 

In the temperature range analyzed, 200–500 °C, the fibers impregnated with primers show another noticeable peak and change in transformation rate. Thus, for fibers impregnated with primer Unicell, this peak is at 388 °C, and the decomposition rate decreases to 1.43%/min. In contrast, for fibers impregnated with primer Soudal, this peak is much more shifted towards higher temperatures and is located at 464 °C. The decomposition rate is slightly slower at 1.29%/min.

Interestingly, such an additional peak is not observed for fibers coated with pMDI. Such a peak for pMDI occurs in the temperature region of 475–485 °C, depending on the type of environment [40]. This peak must, therefore, be masked to some extent by changes occurring earlier. The dynamics of change for the area covering this temperature (440–480 °C) is highly linear (R^2^ = 0.9986), with mass loss decreasing even further towards the end of the analyzed area. As expected, the total mass loss for the analyzed systems is the smallest for the fiber–gypsum composite. Significantly, pMDI limits the decomposition of the hemp fibers up to 600 °C. Furthermore, some differences in the thermostability of the fibers impregnated with the building primers can also be observed. The different courses of thermal decomposition are related to the detailed chemical characteristics of the two products, as the essential compounds are present in both. This interpretation is also supported by the results of tests on the mechanical properties of the produced composites, indicating that primer Soudal has a more favorable effect on the bonding mechanism between the gypsum and the impregnated fibers.

## 4. Conclusions

- Although hemp fibers have a desorption shrinkage similar to that of wood in the length range of approximately 1%, this shrinkage may adversely affect the quality of gypsum boards manufactured with long fibers.
- Hemp fibers are highly hygroscopic, showing a more than 200% water absorption capacity. This can interfere with or make it difficult to maintain the correct water-to-gypsum ratio during the preparation and application of gypsum slurry.
- Using an appropriate fiber impregnation can help reduce water absorption by the fibers, thereby limiting the dimensional changes in the fibers and the negative impact on the water–gypsum balance. The most favorable results were obtained in this respect using Soudal, which reduced the absorbability of the fibers by more than three times.
- Hydrophobically impregnated fibers make it possible to produce gypsum boards with a higher static flexural strength and modulus of elasticity than non-impregnated fibers.
- The introduction of impregnates into the mixing water deteriorates the mechanical properties of the gypsum fiber boards produced in this way.
- The impregnants used in the study not only sedimented well on the fiber surface, blocking access of water to the fiber space, but also, as shown by FTIR analysis, reacted with the free hydrophilic groups of the fibers.
- The TG/DTA analysis results also confirm the changes in the impregnated fibers and plasterboard structure. The impregnated fibers show higher thermal resistance than the non-impregnated fibers.

The tests carried out indicate the great potential of the proposed solution. Although only three impregnates with different chemical compositions were analyzed in this study, and the positive effect of impregnation was not always confirmed in the properties of the boards, the research seems worth continuing. A properly selected impregnate should not only reduce the hydrophilicity of the fibers but also create a structure on their surface that is friendly to the anchoring of the gypsum.

## Figures and Tables

**Table 1:** Technological characteristics of the gypsum used in the study.

Feature	Parameter
Water–gypsum ratio	0.6 L–1 kg
Start of setting	after approximately 3 min.
End of setting	after 30 min.
Exothermic reaction during hardening	max. temp. 50 °C

**Table 2:** Summary of all measurements.

	Pure Fibers	Fibers + Unicell	Fibers + Soudal	Fibers + pMDI	Fibers + Gypsum
Range 50–150 °C (water)					
Weight loss (−) [%]	1.52	1.71	0.3	1.5	2.37
Range 50–150 °C					
T_max1_ (peak temperature) [°C]	-	-	-	-	98
Maximum rate of conversion in T_max_ [%/min]	-	-	-	-	0.47
Range 170–200 °C					
Marked temperature of the beginning of transformations [°C]	184	173	185	177	186
Range 200–500 °C					
T_max2_ (peak temperature) [°C]	349	350	341	348	350
Maximum rate of conversion in T_max1_ [%/min]	7.84	6.73	7.94	5.05	6.79
T_max2_ (peak temperature) [°C]	-	388	464	-	-
Maximum rate of conversion in T_max2_ [%/min]	-	1.43	1.29	-	-
Mass change in the interval [%]	72.07	74.31	75.79	64.21	55.58
Residues from full-rate conversion 35–600 °C					
[%]	24.92	22.4	23.07	30.21	40.65

## Data Availability

The data presented in this study are available on request from the corresponding author.
